# The Contribution of Occult Precipitation to Nutrient Deposition on the West Coast of South Africa

**DOI:** 10.1371/journal.pone.0126225

**Published:** 2015-05-27

**Authors:** Justine M. Nyaga, Jason C. Neff, Michael D. Cramer

**Affiliations:** 1 Department of Biological Sciences, University of Cape Town, Cape Town, South Africa; 2 Department of Biological Sciences, Embu University College, Embu, Kenya; 3 Geological Sciences and Environmental Studies, University of Colorado at Boulder, Boulder, Colorado, United States of America; University of California Davis, UNITED STATES

## Abstract

The Strandveld mediterranean-ecosystem of the west coast of South Africa supports floristically diverse vegetation growing on mostly nutrient-poor aeolian sands and extending from the Atlantic Ocean tens of kilometers inland. The cold Benguela current upwelling interacts with warm onshore southerly winds in summer causing coastal fogs in this region. We hypothesized that fog and other forms of occult precipitation contribute moisture and nutrients to the vegetation. We measured occult precipitation over one year along a transect running inland in the direction of the prevailing wind and compared the nutrient concentrations with those in rainwater. Occult deposition rates of P, N, K, Mg, Ca, Na, Al and Fe all decreased with distance from the ocean. Furthermore, ratios of cations to Na were similar to those of seawater, suggesting a marine origin for these. In contrast, N and P ratios in occult precipitation were higher than in seawater. We speculate that this is due to marine foam contributing to occult precipitation. Nutrient loss in leaf litter from dominant shrub species was measured to indicate nutrient demand. We estimated that occult precipitation could meet the demand of the dominant shrubby species for annual N, P, K and Ca. Of these species, those with small leaves intercepted more moisture and nutrients than those with larger leaves and could take up foliar deposits of glycine, NO_3_-, NH_4_^+^ and Li (as tracer for K) through leaf surfaces. We conclude that occult deposition together with rainfall deposition are potentially important nutrient and moisture sources for the Strandveld vegetation that contribute to this vegetation being floristically distinct from neighbouring nutrient-poor Fynbos vegetation.

## Introduction

The coastal Strandveld vegetation in the Cape Floristic Region (CFR) of South Africa is a relatively dense shrubland containing sclerophyllous and drought deciduous shrubs and low trees [1] occurring on sand dunes that extend up to tens of kilometers inland from the Atlantic Ocean. Unlike other low-nutrient status ecosystems of the CFR, the Strandveld is characterized by comparatively high soil and plant P, base cations, and organic matter, despite having soils that are 96–98% sand with very little clay [2, 3]. In contrast to the Strandveld sands, the recently deposited fore-dune sand nutrient contents are very low. For example, total N, available P, K and C concentrations were 15-, 4.4-, 4- and 10-fold higher, respectively, in Strandveld soils than in adjacent coastal dune sands [4]. The relatively nutrient-rich status of the Strandveld is thus at odds with the high sand content and origins of these soils, and indicates that C and nutrients accumulate in these sands during pedogenesis.

Soils are the combined product of the regional climate, biota, topographic relief, the parent geology and the age of the soil [5]. Additionally, wet and dry deposition of dust and atmospheric nutrients can play a major role in determining soil characteristics [6, 7]. Wet deposition is commonly an important source of nutrients for ecosystems around the world, and especially in coastal areas [8]. In many instances precipitation is transported and deposited horizontally as clouds, mist, drizzle and fog [9, 10, 11, 12, 13]. Sea spray aerosols form the largest component of the marine boundary layer particulate concentrations [14] having relatively high concentrations of some nutrients, especially base cations, N [15, 16], and P [17]. Marine aerosols are produced due to agitation of the surface waters by wind [18] and incorporation into fog allows aerosols to be dissolved or suspended in the fog moisture [14]. This nutrient-rich moist air precipitation, referred to as occult deposition, may be carried onshore by winds and deposited in terrestrial ecosystems where it may form an important nutrient source [9]. Sea spray may also be blown directly off the sea surface onto the shore where it could also be an important nutrient source for the terrestrial ecosystem [19].

Deposition of aerosols provides ecologically significant contributions of P (0.2 kg ha^-1^ a^-1^) and N (2 kg ha^-1^ a^-1^) to lowland Fynbos vegetation within the CFR [20, 21]. Furthermore, inland pans close to Strandveld vegetation have ratios of Cl to Na and Mg that indicate a predominantly marine source for these ions [22]. The high cation content of Strandveld sands has also been speculated to be attributable to marine aerosols [2], although wind-blown terrigenous mineral dust may also contribute Ca, K, P and Fe [23]. Collectively, these prior studies and observations of soil nutrient characteristics in the Strandveld raise the question as to whether deposition is an important component of the nutrient supply to the Strandveld vegetation of the CFR.

In the Strandveld the intensity of herbivory is relatively low [24] and fires are relatively infrequent (2–0.5 per century; [25]). Strandveld grows to approximately 2 m tall [26] and thus annual increases in woody biomass of vegetation that has not been burnt for a long time are small. Therefore losses of nutrients from plants are mainly through above- and below-ground tissue senescence. Although below-ground senescence is likely to be important, this is difficult to assess [27]. The loss of nutrients in leaf litter depends both on the volume of litter and the nutrient concentration of the litter, which is determined by the capacity of the plant for resorption of different nutrients and varies strongly between nutrients and species [28]. Nutrients that are resorbed prior to litterfall are directly available for further growth, whereas nutrients lost in litterfall need to be replaced from the soil. Recycling of nutrients in litterfall requires decomposition over years [29] with potential losses from the ecosystem. Thus, in the short-term, the flux of nutrients in litterfall constitutes a loss from the plant and provides a good approximation for annual plant nutrient demand [30]. In the absence of substantial woody biomass increase, litterfall can also be used as an estimate of net primary productivity [31].

In some Mediterranean coastal ecosystems, including the west coast of South Africa, occult deposition is the primary form of precipitation during summer [9, 32] and may provide an important source of water [33; 34; 11] and nutrients [35, 36, 12]. Plant canopies may intercept some of this precipitation where it coalesces into droplets that fall to the ground as “fog drip” [37; 9] providing moisture and nutrients to the plants through the soil [38]. Leaves may also directly absorb the moisture in occult deposition [39] and take up the nutrients [40, 41]. The capacity of leaves to intercept occult deposition varies with leaf morphology, with small narrow leaves being more common in areas prone to frequent fog [42]. Small leaves intercept more canopy fog than larger leaves [42], and it is possible that this interception contributes to ecosystem nutrients [43]. These observations suggest that occult deposition may provide water and nutrient to some ecosystems, such as the coastal Strandveld.

We hypothesized that the Strandveld vegetation depends on marine nutrient deposition and that, over long periods of time, this source of nutrients has resulted in soil and vegetation nutritional characteristics that are associated with the mainly marine origins of this deposition. To test this we compared the soil and foliar nutrient compositions of Strandveld vegetation in the West coast National Park (South Africa) to the nutrient composition of rainwater and occult deposition measured at the site. We also used litterfall and the nutrient composition of the litter to indicate plant nutrient demand and compared this to the rates of nutrient deposition. To determine whether the foliar properties and canopy architecture of the native vegetation contributed to interception of occult deposition, we evaluated whether leaf size variations of Strandveld species contribute to differential rates of canopy interception of occult water and nutrients.

## Methods

### Study site

**Fig 1:**
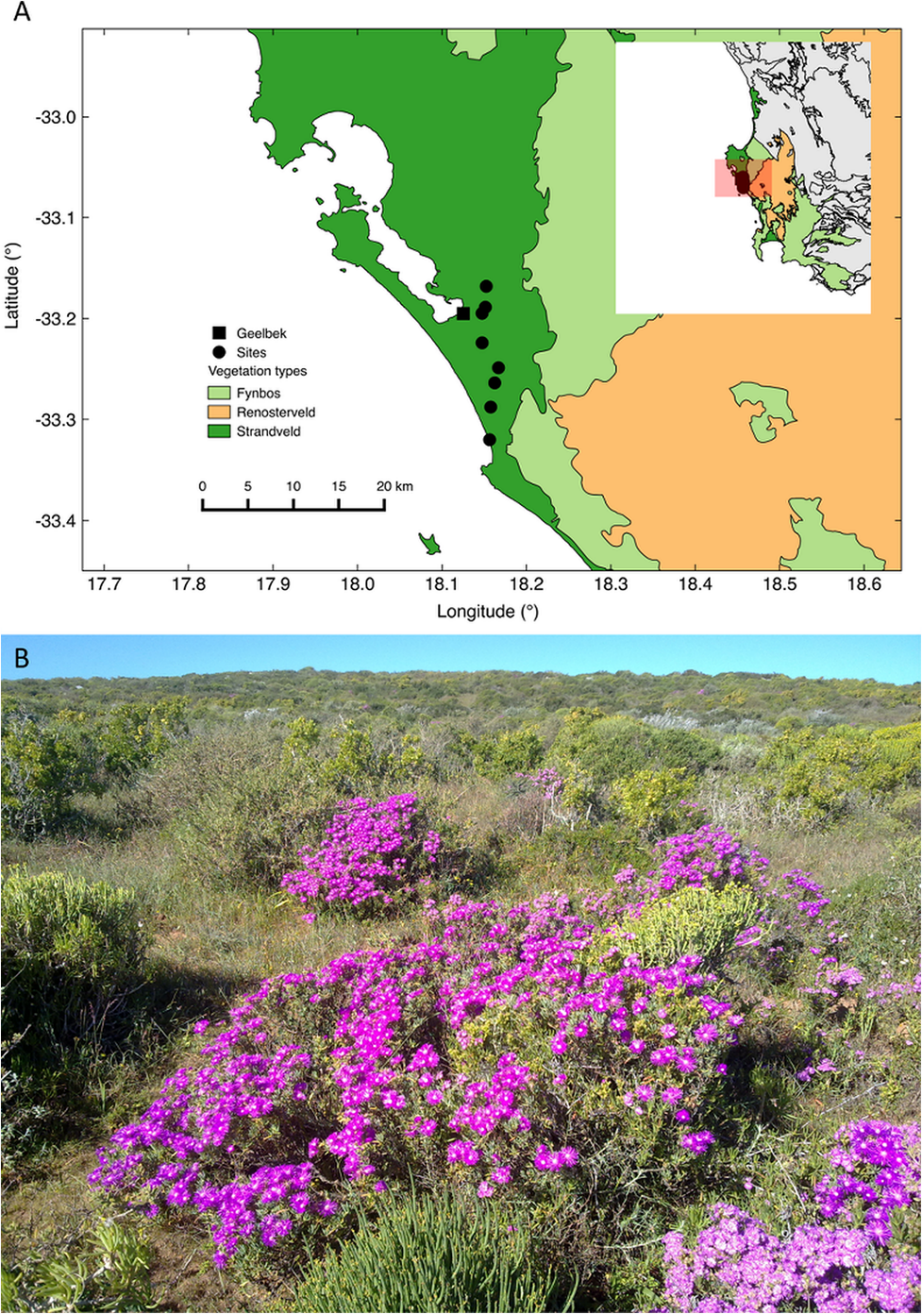
A) Map of study site and B) typical low-stature Strandveld vegetation of the West Coast National Park. The map shows the location of the Geelbek weather station at the West Coast National Park (square symbol) that is 2.2 km from the mid-point of the sample sites (circular symbols). Inset map shows site location within South Africa. The coastline and the regional vegetation types that include Strandveld (study site), Fynbos and Renosterveld are shown [26].

The sampling was carried out in the West Coast National Park (-33.231183°, 18.164156°) on a 17 km migrating dune cordon running inland from the coast in a northerly direction (Fig 1A). The South Africa National Parks provided permission and facilitated the research. The park is approximately 100 km northwest of Cape Town along the Atlantic Ocean coastline. The area has a Mediterranean climate with mild wet winters and hot dry summers, and experiences strong southerly winds for most of the year, but particularly in summer. The autumn and spring seasons are intermediate between winter and summer with respect to rainfall, temperature and wind. The coast is exposed to moderate- to high-wave energy, with 90% of waves having heights of 1–3 m [44].

The geology of the area consists of basement rocks of the Malmesbury formation overlaid by loose sand sculpted into flats, dunes and hollows by the strong southerly winds [45]. The aeolian sands are marine-derived and contain a large proportion of calcareous material [44]. The main vegetation unit in the area is the Langebaan dune Strandveld, which dominates most of the deep calcareous sands and consists mainly of sclerophyllous shrubs (*ca*. 2 m tall, Fig 1B) and annual herbs [26]. The area near the ocean has been extensively invaded by a fast growing alien N_2_-fixing *Acacia cyclops* (not sampled) which co-occurs with the indigenous *Chrysanthemoides monilifera*. Two *Searsia* spp. (*S*. *lucida*, *S*. *glauca*) dominate inland vegetation. Other species common within the study site include *Agathosma imbricata*, *Metalasia muricata* and a native N_2_-fixing species, *Morella cordifolia*. These six species were selected for green leaf and litterfall sampling.

### Precipitation and seawater sampling

Eight different sampling locations were established at varying distances from the beach, (0.1, 3.6, 6.1, 8.0, 10.7, 12.5, 14.6 and 16.8 km) along the dune plume. Collectors (S1 Fig) consisted of a screen (34.3 cm x 64.4 cm) made of 128 nylon lines reaching a height of *ca*. 2 m. The channel at the bottom of the screen was open and could directly intercept precipitation resulting in the collector not differentiating between horizontal precipitation and rain. Thus we refer here to the moisture collected by these contraptions as “horizontal precipitation” (HP), which includes both sources of moisture. The surface area of the collector that was effective for collecting occult deposition was estimated as the sum of the surface areas of all the 128 lines and the channel below them creating a collecting surface of 1554 cm^2^ that drained into a 27 cm^2^ channel. Stainless steel spikes were fitted on top of the screen to prevent birds perching, and consequent contamination. Samples were discarded if there was any evidence of contamination (e.g. bird droppings or insects) on the collectors (one sample was discarded). The collecting channel was connected to a 2 L Schott bottle by a 6 mm ID tube that was looped to form a moisture trap and thus reduce sample evaporation [46]. Rainfall collectors comprising 113 cm^2^ funnel similarly connected to a 2 L Schott bottle were installed with each HP collector and sampled and analyzed as described for HP [47].

The sampling bottles were thoroughly cleaned and rinsed with Millipore water (Elix 20 water system, Merck Millipore, Darmstadt, Germany). A biocide, (200 mg of 2-isopropyl-5-methylphenol) was added to each of the bottles (including blanks) prior to sample collection to minimize microbial degradation of the sample [48]. They were changed monthly for 12 consecutive months (Jan-2011 to Dec-2011). Prior to sample removal, the screen was washed with 50 mL ultra-pure Millipore water using a squirt bottle to wash off dry deposition on the collector and this wash was included in the sample. Sample volumes and concentrations were then corrected for the volume of the water rinse by excluding the rinse volume from the collected volume and correcting the concentration to the collected volume.

Between Aug-2011 and Dec-2011, *ca*. 200 mL of seawater was collected monthly *ca*. 50 m offshore beyond breakers. Clean plastic containers pre-rinsed with Millipore water were used in sampling and biocide added after collection. All samples were stored at 4°C for a maximum of 2 d prior to transfer to 50 mL centrifuge tubes and storage at -20°C. Elemental values of sea water were expressed relative to Na, as has been done previously [49].

### Environmental variables

Daily rainfall, and hourly wind direction and wind speed data were obtained from the Geelbek weather station located within the study area (West Coast National Park management), 2.2 km west of the 14.6 km sampling site. Wind speed and direction was summed cumulatively for calculation of the cardinal and intercardinal wind speed/directions for the wind roses.

### Leaf litter and soil sampling

At the eight sampling locations, sampling plots measuring 50 m^2^ were established in which at least three of the selected study species were present. In each plot, we collected litterfall material every month for 12 months (Jan-2011 to Dec-2011), and mature fully expanded green leaves in Nov-2011. At least three replicates of each of the six study species (*M*. *cordfolia*, *C*. *monelifera*, *S*. *glauca*, *S*. *lucida*, *A*. *imbricate*, *M*. *muricata*), distributed across the 8 locations, were selected and tagged for repeat sampling. Litter traps made from a nylon mesh (0.2 mm) measuring 0.5 x 0.5 m and 0.1 m deep were placed under each of the selected shrubs at the beginning of the experiment. After clearing away any leaf litter, three replicate soil surface cores (<0.3 m depth) were taken from each sampling location in Nov 2011 using a soil auger (10.2 cm internal diameter), and stored in plastic bags at 4°C prior to analysis.

### Nutrient washing from leaf surfaces

Terminal twigs (0.05–0.1 m length) were cut from the six dominant woody species at the study site in Nov-2011, bagged in plastic and stored in a cooler box. Leaves were then washed with 50 mL of Millipore water within the bag they were collected in, and the wash-samples stored at -20°C prior to analysis.

### Leaf and canopy water holding capacity

Potted plants (n = 3) of each of the six species were obtained from the Kirstenbosch nursery (Cape Town) and kept in a greenhouse for 5 d. The plants were rooted in mixture of sand and compost (ratio of 1:1) in 2 L plastic bags. Three mature green leaves from the sampled portions of each of three replicate plants were weighed, their dimensions taken and areas determined using a LI-3000 Area Meter (LICOR Lincoln, NE USA). Leaf dimension was determined as the diameter of the largest circle that could fit in the leaf perimeter [50]. The leaves were dipped in water and reweighed to determine their water holding capacity. Leaf water holding capacity was expressed as the difference between the fresh weight and wetted weight and expressed per leaf area (kg m^-2^).

The canopy water holding capacity was determined by gradually wetting a pre-weighed intact branch obtained from each plant to saturation in a simulated horizontal precipitation experiment inside a wind tunnel (1.5 m length x 0.3 m diameter). The branches were suspended *ca*. 0.7 m from a fan generating a measured wind speed of *ca*. 10 m s^-1^, which was the maximum measured hourly wind speed at the study site in 2011. Thus the measured foliar interception of moisture and nutrients is likely the lower limit of what could be intercepted, although wind gusts must exceed the maximum hourly average. A mist stream generated using a pressurized sprayer was introduced between the fan and the suspended branches *ca*. 0.2 m from the fan. Branches were saturated with water and reweighed, and the difference between the fresh weight and wetted weight determined. The leaves were removed and the total leaf area measured using LI-3000 Area Meter. The water holding capacity of the leaf surface was expressed per canopy area (kg m^-2^).

### Foliar nutrient uptake

^15^N-labeled NaNO_3_, NH_4_Cl and glycine (98% atom, Sigma-Aldrich, St. Louis Missouri, USA) were separately dissolved in 100 mL of water to make 0.22, 0.35 and 0.25 mM solutions, respectively, and applied to replicates of separate young fully expanded leaves of potted plants (n = 3). The label was applied by covering the leaf with a blotting paper soaked in a solution of the label for 6 h. Lithium chloride (0.089 mM) was applied in a similar procedure. Leaves were harvested and rinsed thrice in 1 mM CaCl_2_ to remove excess label, and then oven dried at 60°C for 48 h and milled to fine powder (Mixer Mill MM400, Retsch GmbH, Haan, Germany). Samples (2.8–3.0 mg) of the ground sample were weighed in 5 × 9 mm tin capsules (Santis Analytical AG, Teufen, Switzerland) and analyzed for N isotopes by combustion methods [51] using a Thermo Flash EA 1112 series elemental analyzer (Thermo Electron Corporation, Milan, Italy). The stable N isotopes (δ^15^) were measured using a Delta Plus XP isotope ratio mass spectrometer in the Archeometry laboratory at the University of Cape Town. The ^15^N enrichment was expressed as the difference between the δ^15^N values measured in the treated leaves compared to unlabeled leaves. Foliar Li concentration was measured as described for leaf sample analysis below, and the values reported on a dry-weight basis.

### Leaf and soil analysis

Leaf and soil samples were oven dried at 60°C for 48 h. The total mass of collected leaf litter was recorded monthly. Leaf litter and green tissue collected in Nov-2011 were milled to fine powder (Mixer Mill MM400) for chemical analysis. Soils were sieved (2 mm) and a sub-sample ground in a mortar for chemical analysis. Vegetation and soil samples were chemically digested following methods used in [52]. All samples were analyzed for elemental composition by Inductively Coupled Plasma Atomic Emission Spectroscopy (ICP-AES, ARL 3410+) and Inductively Coupled Plasma Mass Spectroscopy (ICP-MS, Perkin Elmer Elan DRC-E, Waltham, Massachusetts, USA) at the Laboratory for Environmental Geoscience at the University of Colorado. Two bedrock standards (Silver Plume Granodiorite and Hawaiian Basalt) were included in each extraction simultaneously with the sample to check for any analytical uncertainty (typically < 8%). Total N was analyzed with a TOC/TN high temperature combustion analyzer (Shimadzu, Kyoto, Japan).

### Water sample analysis

All precipitation and leaf wash samples were analyzed for NO_3_^-^, NH_4_^+^ and PO_4_^3-^ using colorimetric procedures. Analysis for NH_4_^+^ followed the phenol-hypochlorite method [53], and NO_3_^-^ analysis the vanadium chloride method [54]. Dissolved organic nitrogen (DON) was calculated as the difference between total N and the sum of NH_4_^+^ and NO_3_^-^. The malachite green oxalate method [55] was used to determine the concentration of PO_4_^3-^, but other phosphates can be hydrolyzed to orthophosphate during this analysis. The measured P is thus reported as soluble reactive P (SRP). A 1500 Multiskan spectrum plate reader (Thermo Electron Corporation, Vantaa, Finland) was used to determine sample absorbencies. The detection limit for each analysis was determined by dividing the standard error of the standard sample absorbencies by the slope of the standard curve [56]. Nutrient concentrations below the detection limit were assigned a value of one half the detection limit. The detection limits for NH_4_^+^, NO_3_^-^ and SRP were determined to be (mg L^-1^) 0.0458, 0.0251 and 0.01 respectively. Most NH_4_^+^ values were found to be below the detection limit. Five duplicate runs of a randomly selected sample produced analytical variances of 7.8%, 5.0% and 4.3% for NH_4_^+^, NO_3_^-^ and SRP analysis, respectively.

Precipitation samples were analyzed for total P, Na, Mg, Ca, Si, K, Fe and Al using ICP-AES/MS. Values that were below the detection limits (mg L^-1^: 0.077, dissolved P; 0.095, Na; 0.005, Mg; 0.052, Ca; 0.141, K; 0.029, Si; 0.002, Mn; 0.009, Fe; 0.012, Al) were assigned a value of one half the detection limit. A standard sample was analyzed alongside the water samples yielded analytical variances of 0.5% for dissolved total P, 3.37% for Mg, 1.43% for Ca, 3.19% for Na, 1.74% for K, 4.57% for Si, 4.56% for Mn, 4.54% for Fe, and 4.59% for Al (n = 6). Total N was analyzed with a TOC/TN (Shimadzu) high temperature combustion analyzer with detection limit of 0.05 mg L^-1^.

### Data analysis

The coastal site (0.1 km) was excluded from most analyses, apart from the analysis of variations in deposition with distance from the ocean, due to the very high concentrations of nutrients (S1 Table). Where appropriate, data were analyzed using Student’s t-tests, one-way ANOVA or ANCOVA (categorical variable = season; continuous variable = distance from ocean) followed by *post-hoc* Tukey tests (Statistica ver. 8, StatSoft, Inc., Tulsa, OK, USA), as detailed in table and figure captions. Where no interactions were found between predictor variables, averages were reported for both season and distance from the ocean.

## Results

### Nutrient deposition

**Fig 2:**
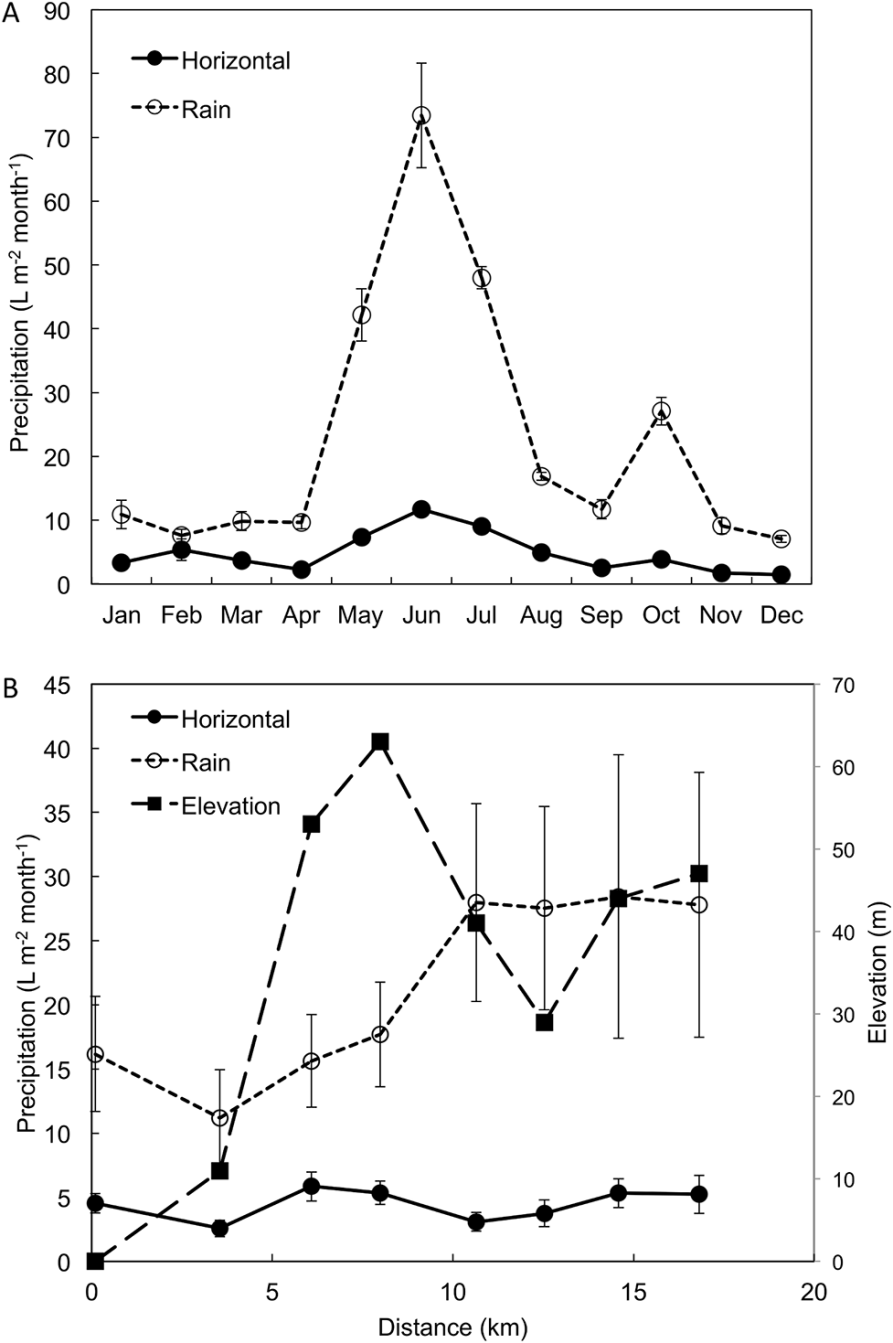
Rain and horizontal precipitation between Jan and Dec 2011 expressed as monthly volume collected per surface area of the respective collectors. A) The annual variation (mean ± SE; n = 7 sites, excluding the site closest to the coast) and B) the variation in annual average precipitation (mean ± SE; n = 12 months) along the transect from the coast inland. The elevation of each of the collection points is also shown in panel B.

**Fig 3:**
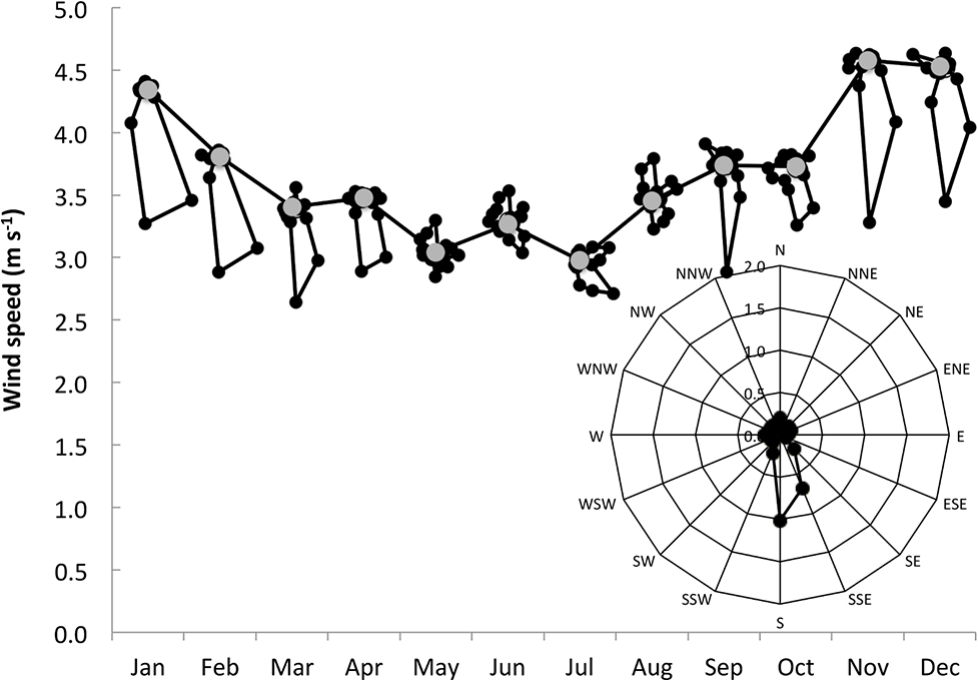
The variation in monthly average wind speed from the Geelbek weather station at the West Coast National Park that is 2.2 km from the mid-point of the transect (Fig 1). The wind-roses for each month are shown with their center points being the average wind speed (grey point). The inset wind-rose shows the average annual wind speed and direction on the same scale as used for the monthly data.

The design of the HP collectors was intended to mimic the potential trapping of wind-blown aerosols, fog and horizontal rainfall by vegetation. The design was such that wind blown rain was included in the HP samples, and thus HP represents a summation of the deposition from both rain and other forms of moisture. The volumes collected in the rainfall and HP collectors both peaked in the winter months of May–Jul (Fig 2). The volume collected by the HP collectors was lower than the rain volume when expressed per collector surface area. The rain volumes were larger inland than at the coast, whereas HP variations with distance from the coast were small. Nevertheless, variation in HP may be partially associated with the elevation of the sampling sites with lower elevation sites away from the coast receiving less HP than those at higher elevations (S2 Fig). Stronger winds occur in summer than other seasons (Fig 3). The prevailing wind direction at this site is dominated by southerly winds that blow off the Atlantic Ocean, but the southerly component abates in winter when there is a stronger northerly component to winds. As a consequence of the focus of rainfall in the mid-winter period and wind in the mid-summer period, we reported both annual and seasonal averages of deposition.

Over the course of an annual sampling cycle, the HP collectors collected substantially higher concentrations of N and K than the rainfall collectors (Table 1). Deposition flux was higher in HP than in rainfall for N, Mg, Na and K, but lower in SRP, Ca, Si, Mn and Al (Table 1). The annual concentrations (Table 1) were influenced by a high degree of seasonality in the concentrations of some nutrients in HP collectors (Table 2). Total N, NO3—DON, total P, SRP, Mg, Ca, Na, K, Si, Mn all had significantly higher concentrations in summer, compared to winter, when HP was highest (Table 2, Fig 2).

**Table 1:** Monthly concentrations and deposition fluxes in horizontal precipitation (HP) and rain.

	Elemental concentration (mg L^-1^)			Deposition flux (mg m^-2^ month^-1^)		
Element	HP	Rain	*P*	HP	Rain	*P*
total N	27.1±2.1	1.4 ± 0.2	**<0.001**	85 ± 8	32 ± 4	**<0.001**
NO_3_^-^	3.35±0.45	0.5 ± 0.08	**<0.001**	8.56 ± 1.08	3.83 ± 0.41	**0.001**
DON	22.8±1.9	1 ± 0.2	**<0.001**	74 ± 6	25 ± 4	**<0.001**
total P	0.43±0.07	0.32 ± 0.2	0.622	1.27 ± 0.13	1.44 ± 0.19	0.467
SRP	0.14±0.03	0.16 ± 0.04	0.650	0.29 ± 0.04	0.84 ± 0.09	**<0.001**
Mg	76±16	42 ± 25	0.268	193 ± 34	66 ± 9	**0.003**
Ca	41±4	112 ± 64	0.287	111 ± 12	165 ± 13	**0.006**
Na	640±141	370 ± 229	0.334	1604 ± 303	572 ± 88	**0.006**
K	23.3±4.1	4.4 ± 0.8	**<0.001**	60 ± 9	30 ± 4	**0.009**
Si	0.7±0.2	1.8 ± 1.2	0.386	1.57 ± 0.42	2.59 ± 0.22	**0.046**
Mn	0.05±0	0.11 ± 0.07	0.356	0.145 ± 0.017	0.23 ± 0.03	**0.041**
Fe	0.06±0.02	0.02 ± 0.00	0.185	0.187 ± 0.046	0.31 ± 0.05	0.078
Al	0.09±0.03	0.16 ± 0.1	0.491	0.29 ± 0.06	0.59 ± 0.07	**0.005**

Depositional fluxes are the product of the total measured monthly precipitation volume, corrected for the wash water volume added, and concentration of the nutrient in the sample. Annual values (Jan-Dec 2011) and across all sites (mean ± SE, n = 7), but excluding the site closest to the coast (S1 Table). Significant differences (*P* < 0.05) between rain and HP concentrations were determined using Student’s t-tests.

**Table 2:** Seasonal (i.e. over 3 months) horizontal precipitation (HP) volumes and elemental concentrations.

	Autumn	Winter	Spring	Summer	*P*
HP vol.	4.6±0.6a	8.9±0.8b	2.4±0.2a	2.8±0.5a	**<0.001**
total N	26±3b	11±1a	32±3b	33±5b	**<0.001**
NO_3_^-^	1.12±0.06a	1.91±0.65a	4.27±0.6ab	5.38±1.79b	**0.003**
DON	25±3b	9±1a	26±3b	26±5b	**<0.001**
total P	0.36±0.05b	0.15±0.02a	0.42±0.05b	0.65±0.09c	**<0.001**
SRP	0.06±0.01a	0.03±0.01a	0.12±0.02a	0.33±0.13b	**0.002**
Mg	39±4ab	20±3a	88±12bc	121±30c	**<0.001**
Ca	29±3ab	13±2a	43±5b	75±14c	**<0.001**
Na	289±31a	166±24a	764±95b	1015±260b	**<0.001**
K	11±1a	6±1a	27±3b	39±8b	**<0.001**
Si	0.28±0.04a	0.15±0.02a	0.63±0.13ab	1.1±0.29b	**<0.001**
Mn	0.051±0.008b	0.016±0.003a	0.041±0.005ab	0.092±0.016c	**<0.001**
Fe	0.08±0.02b	0.01±0a	0.05±0.02ab	0.03±0.02ab	**0.027**
Al	0.15±0.03b	0.02±0a	0.07±0.02ab	0.05±0.02a	**0.001**

Precipitation volume (L m^-2^ month^-1^) and averaged elemental concentrations (mg L^-1^ month^-1^) of nutrients measured in HP collected for 12 consecutive months (Jan–Dec-2011) in the study area. The monthly data was grouped into four climatically distinct seasons of three months each: Autumn (Mar, Apr and May), winter (Jun, Jul and Aug), spring (Sep, Oct and Nov) and summer (Dec, Jan and Feb) and averaged across the sites (mean ± SE, n = 7), but excluding the site closest to the coast (S1 Table). Different letters indicate significant seasonal differences (*P* < 0.05, bold text) determined using ANCOVA (categorical variable = season; continuous variable = distance from ocean) followed by post-hoc Tukey tests. There were no significant distance x season interaction effects on any of the nutrients except SRP.

**Fig 4:**
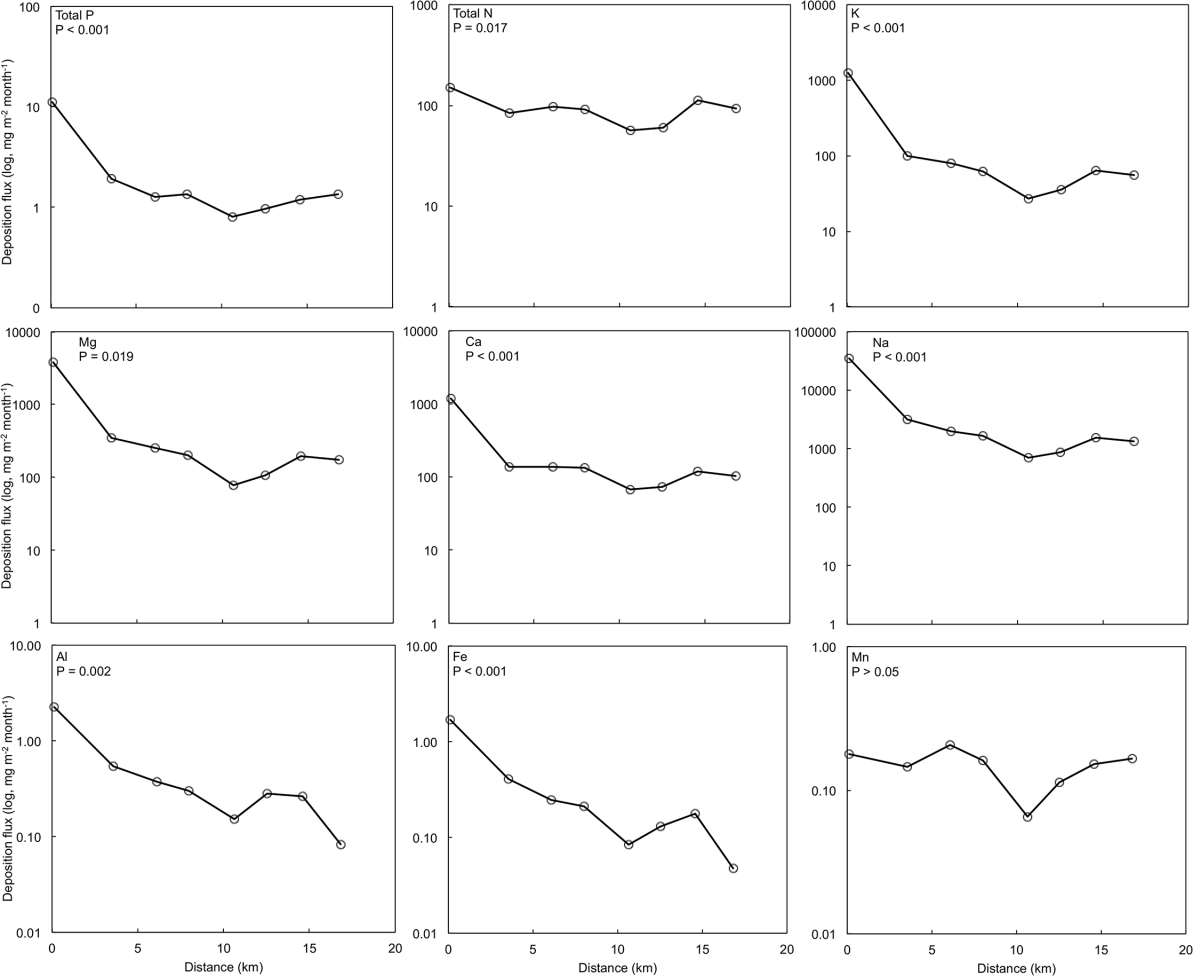
Deposition rates (log, mg m^-2^ month^-1^) of elements measured in horizontal precipitation (HP) at various distances from the ocean (n = 8 sites). Data was collected between Jan-Dec 2011. All elements except Mn declined significantly (*P* < 0.05) with distance inland. Significance was assessed using ANCOVA (categorical variable = season; continuous variable = distance from ocean).

The site immediately adjacent to the beach (0.1 km inland) was particularly heavily influenced by deposition, having higher concentrations of all elements apart from NO_3_^-^ (S1 Table) and was thus excluded from statistical analyses in which the entire transect was considered. Distance from the coast also played a role in determining HP deposition rates of all measured elements, apart from Mn, which was at very low concentrations across all sites (Fig 4). Along the transect of sites inland from the ocean, but excluding the site closest to the ocean, Mg, K, Na, and Ca deposition all declined significantly, whereas there were no significant changes in deposition of the other elements with distance from the ocean when excluding the coastal site. Deposition rates of several elements 10.65 km inland were low. This was associated with lower volumes of HP, possibly arising from differences in site topography (S2 Fig) and thus local reductions in HP (Fig 2).

All elemental molar concentration ratios to Na, except those of SRP and Si to Na, varied significantly through the year (Table 3). The ratios were generally low (expressed as mmol mol^-1^) indicating predominance of Na in HP. Among the components measured, total N, DON and Mg had relatively high values (i.e. > 50 mmol mol^-1^ Na). Seasonal differences in the ratios were relatively small and not consistent between the nutrients. Comparison of the annual average ratios with those of seawater revealed that ratios of Mg, Ca and K to Na were similar to those of seawater (Table 3), but that ratios of other elements (e.g. N, P, Mn, Fe, Al) to Na were orders of magnitude higher than those in seawater. For example total N:Na and total P:Na were 76- and 46-fold greater in HP than in seawater, respectively. There were also significant correlations between the concentrations of Na and base cations in HP (Mg, R^2^ = 0.997, P < 0.001; Ca, R^2^ = 0.82, P < 0.001; K, R^2^ = 0.98, P < 0.001; data not shown).

**Table 3:** Seasonal variations and annual concentration ratios relative to Na in horizontal deposition and in seawater.

Ratio	Autumn	Winter	Spring	Summer	*P*	Annual	Seawater
total N	159±20b	160±38b	77±6a	103±21ab	**0.029**	122±12	1.6
NO_3_^-^	7.9±1.1ab	15.1±3.2b	11.4±2.2ab	6.3±1.2a	**0.043**	10.4±1.1	0.056
DON	150±19b	138±33b	61±4a	92±22ab	**0.013**	108±11	1.4
total P	0.99±0.13b	0.85±0.12b	0.44±0.03a	0.77±0.12ab	**0.002**	0.74±0.06	0.016
SRP	0.15±0.03a	0.22±0.07a	0.11±0.02a	0.25±0.05a	0.098	0.18±0.02	0.004
Mg	131±5b	115±3a	108±3a	116±7ab	**0.002**	117±2	117
Ca	58±4b	50±5b	34±2a	57±7b	**0.001**	49±2	34
K	23±1a	23±0a	21±0a	27±2b	**<0.001**	23±1	33
Si	0.81±0.06a	0.91±0.11a	0.65±0.07a	0.9±0.09a	0.081	0.8±0.04	0.27
Mn	0.072±0.006c	0.047±0.009b	0.025±0.003a	0.053±0.006bc	**<0.001**	0.048±0.004	0.004
Fe	0.16±0.04b	0.03±0a	0.02±0.01a	0.01±0a	**<0.001**	0.06±0.01	0.003
Al	0.57±0.14b	0.15±0.03a	0.08±0.02a	0.04±0.01a	**<0.001**	0.21±0.04	0.00009

The ratios are expressed as mmol mol^-1^ Na (mean ± SE; n = 7), but excluding the site closest to the coast (S1 Table). Different letters indicate significant seasonal differences (*P* < 0.05, bold text) determined using ANCOVA (categorical variable = season; continuous variable = distance from ocean) followed by post-hoc Tukey tests. There were no distance x season interaction effects on any of the nutrients except DON.

### Soil nutrient concentrations

Concentrations of total P, Ca, Mg and Na in soils were high (Table 4), while total N and Fe were low, relative to other soils in the CFR [57]. Because of the high Na concentration in Strandveld soils, elemental ratios to Na (as an indicator of marine influences) were much lower in Strandveld than Fynbos or Renosterveld soils, apart from the ratios for Ca and total P, which were strongly influenced by the accumulation of these in the Strandveld soils. The elemental ratios in Strandveld soils relative to Na were higher than those in HP, largely because soil Na was not as big a component of the cations as it was in HP. The Strandveld soil cation concentration is strongly influenced by high concentrations of Ca.

**Table 4:** Soil elemental concentrations and concentrations ratios to Na in soils from the Strandveld study area compared to values averaged for Fynbos and Renosterveld [57].

	Concentration (mg g^-1^)			Ratio to Na (mmol mol^-1^)		
Nutrient	Strandveld	Fynbos	Renosterveld	Strandveld	Fynbos	Renosterveld
total N	0.21±0.04	1.66	1.20	1342±269	53844	24235
total P	1.5±0.05	0.25	0.28	3012±176	3652	2536
K	0.15±0.0042	0.09	0.15	331±33	1087	1068
Ca	53.0±2.9	1.5	2.1	101965±1795	16663	14802
Mg	1.1±0.09	0.3	0.6	1910±85	5870	6419
Na	0.54±0.03	0.05	0.08	-	-	-
Mn	0.006±0.002	0.065	0.051	14±1.9	539	260
Fe	0.52±0.020	4.8	0.3	1229±187	38867	1492
Al	0.50±0.048	-	-	1325±279	-	-

Values for Strandveld are the mean ± SE (n = 8 sites).

### Leaf litter

The indigenous legume *M*. *cordifolia* and the non-legume *C*. *monilifera* had higher rates of litterfall than the other indigenous species (S3 Fig). The concentrations of nutrients in foliage (expressed on the basis of dry weight) were only assessed during Nov (2011) representing the nutrient status at the end of the growing season, prior to the onset of summer drought. The concentrations of nutrients remaining in senesced foliage as a proportion of that in green tissue was generally small (all < 28%), except for N of which 63% (averaged across species, and 62.8% excluding the legume) was retained in senesced foliage (S2 Table). The estimated proportion of annual litter nutrient loss that could be supplied through HP was calculated as the ratio of estimated plant nutrient demand to the estimated annual flux of the same nutrient in HP. Annual HP nutrient input was close to plant N and P demand and in excess of estimated nutrient demand for K and Ca (Table 5).

**Table 5:** The overall amount of leaf litterfall at the study site averaged across species and the estimated proportion of annual litterfall nutrient loss that could be offset by horizontal precipitation (HP).

	5% Percentile	Mean ± SE	95% Percentile
Litterfall amount (g m^-2^ annum^-1^)	50	185±12	343
% Contribution of deposition in HP			
total N	52	156±24	465
total P	29	92±10	262
K	743	2339±457	7734
Ca	206	692±72	1813

Shown are the 5% percentiles, mean ± SE (n = 7 sites, i.e. excluding coastal site) and 95% percentiles of dry leaf litterfall rates and potential percentage contribution of HP deposition to annual nutrients lost in leaf litter across the six species sampled at the study site. Since not all species were present at each site, leaf litter nutrient content (i.e. leaf litter mass per m^2^ multiplied by litter nutrient concentration) was averaged across all species (S2 Table) and compared to the annual deposition of nutrients.

### Foliar nutrient interception and uptake

**Fig 5:**
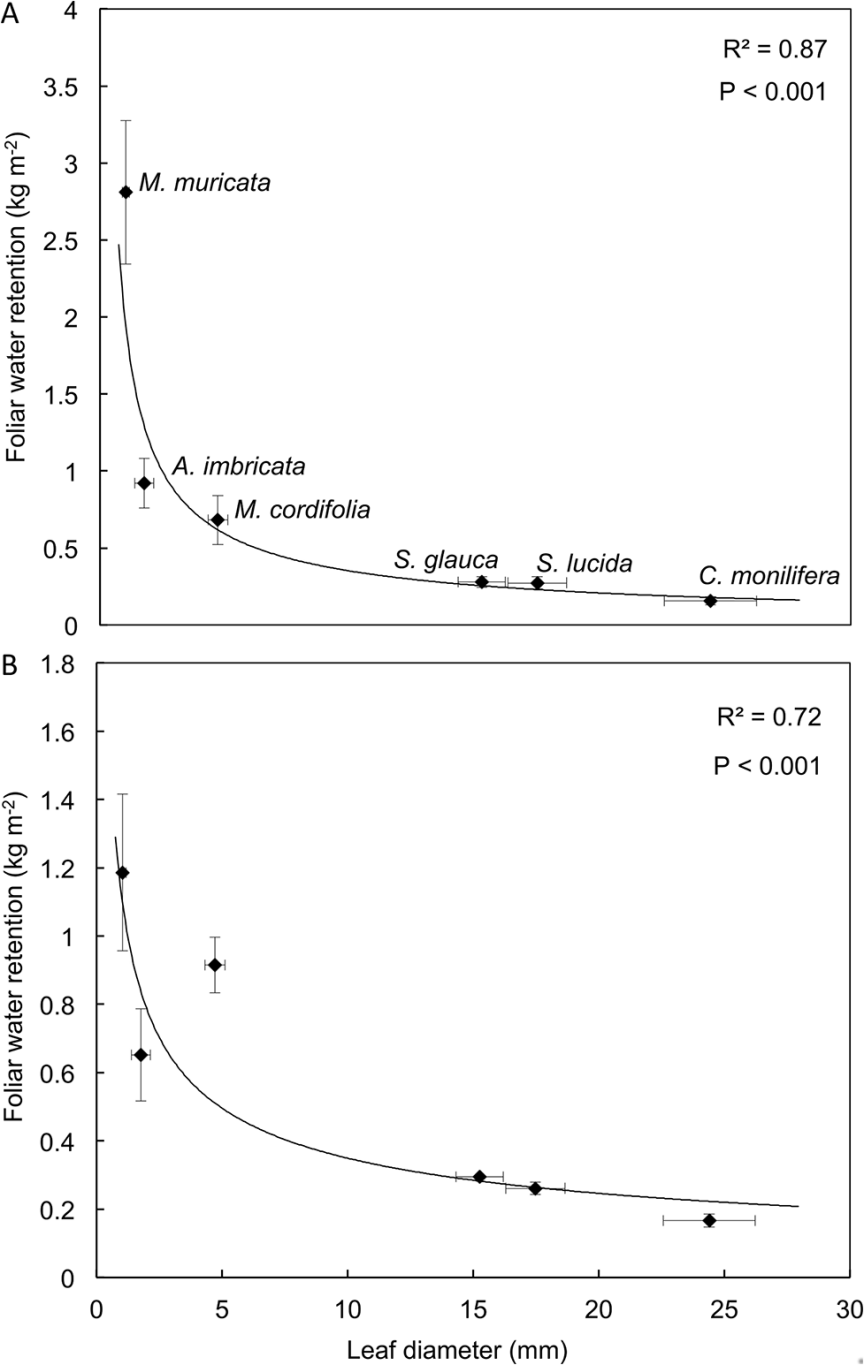
The variation in the amount of water (kg m^-2^) held on A) individual leaves and B) branches of the various species with leaf diameter (LD). Points represent mean ± SE (n = 3). Individual leaves were wetted by dipping them in water, whereas branches were wetted in a simulated horizontal precipitation experiment in a wind tunnel at a 10 m s^**-1**^ wind speed until saturation. The relationship between water held on individual leaves and by branches with LD were fitted by the equations 1.99 × LD^**-75**^ and 1.12 × LD^**-50**^, respectively.

**Fig 6:**
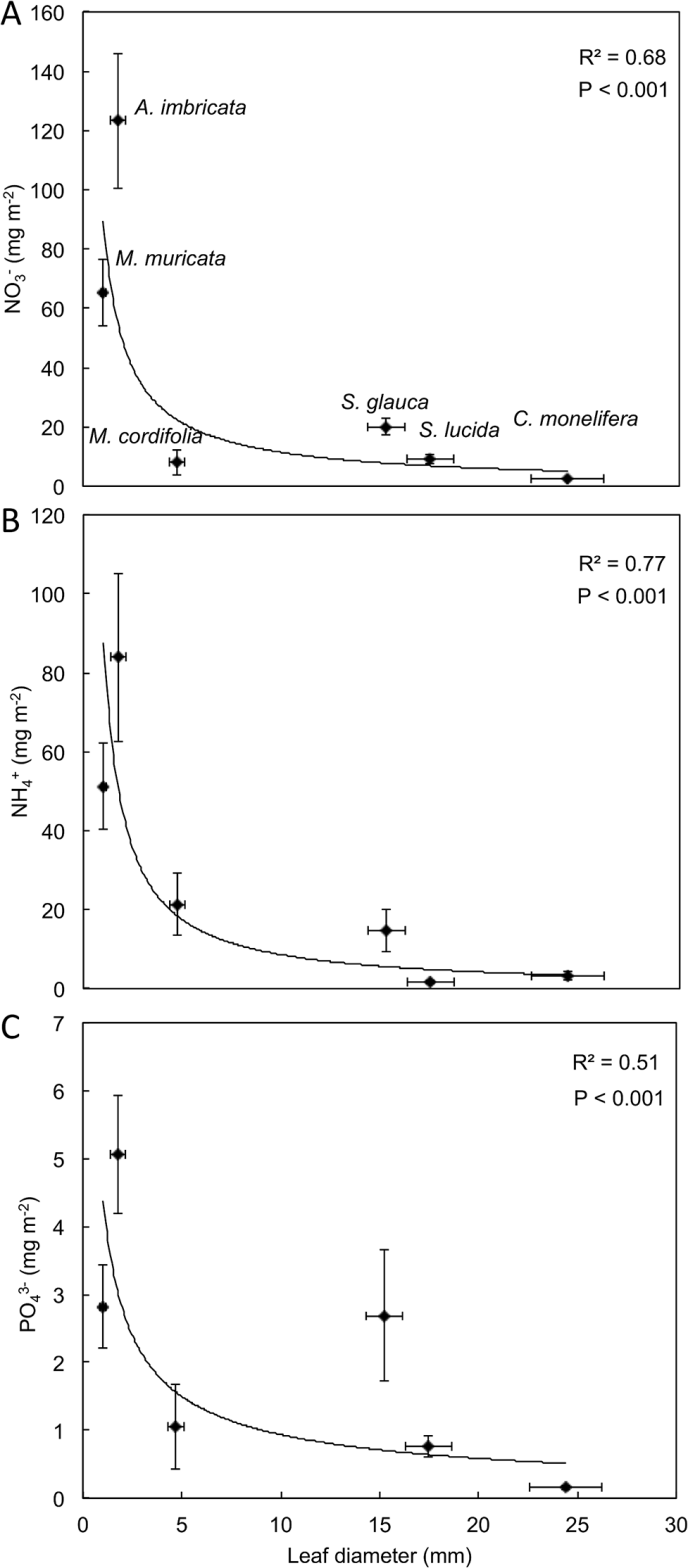
Concentrations expressed per leaf surface area of A) NO_3_^-^, B) NH_4_^+^ and C) PO_4_^3-^ on leaves of various diameters (LD). Points represent mean ± SE (n = 3). The nutrients were measured in Millipore water used to rinse leaves off the various plant species collected from the field in Nov 2011. The relationship between NO_3_^-^, NH_4_^+^ and PO_4_^3-^ and LD were fitted by the equations 91.57 × LD^-0.91^, 90.05 × LD^-1.02^ and 4.46 × LD^-0.07^, respectively.

Plant leaf size and canopy structure were closely associated with both water (Fig 5) and nutrient (Fig 6) interception. The water holding capacities of both leaves and canopies were strongly influenced by leaf diameter (i.e. size), with larger leaves retaining less moisture, expressed per leaf area (Fig 5). Individual leaves had a higher water holding capacity than the canopy, especially for the smaller leaves, indicating that the dense packing of smaller leaves may have partially limited water retention. Water holding capacities of individual leaves and canopies followed similar logarithmic trajectories. The smallest leaves also had the largest amount of NO_3_^-^, NH_4_^+^ and PO_4_^3-^ on leaf surfaces (Fig 6). All species absorbed ^15^N–compounds in the form of glycine, NO_3_^-^, NH_4_^+^ when these were applied to the leaves (Table 6). These plants also took up Li through foliar surfaces. Compared to the other species, *S*. *glauca* and *S*. *lucida* took up the smallest amount of glycine, NO_3_^-^ and Li.

**Table 6:** The uptake of ^15^N-containing compounds and Li supplied to leaves of Strandveld species.

Species name	Glycine Δ^15^N (‰)	Nitrate Δ^15^N (‰)	Ammonium Δ^15^N (‰)	Li (mg g^-1^)
*M*. *muricata*	4.0±0.28c	5.4±0.57a	0.70±0.20a	6.4±2.0b
*A*. *imbricata*	4.5±0.28c	6.9±0.44a	1.0±0.24a	5.2±1.3ab
*M*. *cordifolia*	2.2±0.25b	3.7±0.22ab	2.2±0.083ab	6.4±1.2b
*S*. *glauca*	0.90±0.23ab	2.8±1.1bc	3.3±0.19bc	0.34±0.018a
*S*. *lucida*	0.82±0.37a	1.4±0.22a	1.2±0.12a	0.39±0.060a
*C*. *monilifera*	1.6±0.20ab	4.0±0.82c	3.9±1.0c	0.44±0.16a

Values are the mean ± SE (n = 3) leaf N-isotope enrichments and foliar concentrations of Li after supply of ^15^N-glycine ^15^NO_3_^-^, ^15^NH_4_^+^ and LiCl, respectively. The increase in δ^15^N values of the treated leaves is shown relative to that of unlabeled control leaves (Δ^15^N = δ^15^Ntreated—δ^15^Ncontrol). The different letters represent significant differences between species determined by Tukey post-hoc test following a one-way ANOVA.

## Discussion

Several lines of evidence support the hypothesis that the Strandveld vegetation receives nutritional inputs from predominantly marine-derived deposition. The deposition is unlikely to result from anthropogenic nutrient sources, given the limited industrial sources of aerosols and nitrogen oxides in the region [47], strong on-shore prevailing winds and that the deposition was chemically complex and contained several cations unlikely to be of industrial origin. The Strandveld vegetation is relatively dense with aboveground biomass of up to 18.1 tons ha^-1^ in comparison to Fynbos vegetation of the CFR (6.5–11.6 tons ha^-1^; [58]). The largely marine-derived aeolian sands have been leached of carbonate [2] leaving nearly pure (99.7% SiO_2_) fine quartz sands in the surface soils [59] which should, in principle, leave the soils with few nutrients to support plant growth. However, despite the tendency toward low nutrient content in the soils of this region, we measured high soil total P, Ca, K, Mg, N and Mn concentrations in these Strandveld soils compared to other parts of the CFR (Table 4) and in comparison to dune sand [4]. P enrichment in these soils may result from long-term P deposition, with P being bound to Ca to form Ca-P [60], thus stabilizing P against leaching. Although soil Na concentration was high in Strandveld soils relative to other regional soils, the ratios of nutrients other than K to Na in the Strandveld soils were much higher than in HP. This is possibly the consequence of leaching of Na and K from the soils. Lower soil total N in Strandveld compared to other CFR soils may also reflect the relatively recent deposition of sands, which, as in chronosequences [61, 62], may accumulate N as C accumulates in the soil. The smaller concentration of Fe in Strandveld compared to other CFR soils is probably due to the high sand and low clay content of the Strandveld soils [2].

Although the Strandveld receives most (60%) of its rainfall May–Jul, a variety of additional mechanisms deliver moisture and nutrients to the ecosystem. The rough seas and extensive wave action in the region generate marine aerosols [63]. Summer fogs form due to cooling effect of the cold Benguela upwellings and are advected inland [64, 65], accounting for a substantial proportion annual precipitation [32]. In summer a low altitude haze frequently extends several kilometers inland from the ocean (personal observation). This occult precipitation delivers moisture to both conventional rainfall collection instruments and to those designed to capture HP. Although we cannot estimate the absolute fraction of precipitation that is related to occult precipitation, the relative contribution of moisture shifts seasonally with a greater fraction of moisture derived from HP in summer than in winter.

The variation of nutrient concentrations in HP across seasons and with distance from the ocean provides some insight into the potential pathways of deposition. The significant decline in deposition rates inland from the ocean and very high concentrations (particularly of Na) immediately adjacent to the ocean and correlations of Mg, Ca and K with Na provide a strong indication that the presence of these elements in HP is related to marine aerosol production. The higher ratios of total N:Na and total P:Na in HP than in seawater was consistent across all sampling sites and may indicate either that there are other terrestrial or marine inputs of N and P. We suggested previously that N in rainfall collected at this site was from non-industrial sources and that P in rain could be from terrestrial (dust and/or mining) or marine sources [47]. Here we argue that HP is enriched with marine-derived N and P relative to seawater on the basis of three lines of evidence. Firstly, at the site 0.1 km from the ocean, N and P were much higher than at other points along the transect. Secondly, dissolved organic N is common in marine aerosols, although concentrations are typically much lower than those observed in this study [66]; the proportion that DON constituted of total N in HP (81%) is similar to that measured in rain water (84%) at this site, and within the range (15–97%) measured in cloud water at a remote coastal site in Chile [12]. Thirdly, DON and SRP concentrations in HP are highest during the months when HP is a larger fraction of total precipitation and southerly on-shore winds are strongest. We speculate that the source of the DON and SRP is organic rich aerosols derived from algae during sea-spray aerosol formation [67, 68]. This is also consistent with (personal) observations of wind blown sea foam deposited several kilometers inland. This foam has been collected along the west coast of South Africa and found to be derived from coastal kelp forests and contains large numbers of bacteria, 21% (w/w) protein, 6.1% lipid (triglycerides) and 2.4% carbohydrate [67, 69]. Foam therefore has high concentrations of N and phospholipids [70] and represents a potential source of high concentrations of N and P in HP.

Another line of evidence for the importance of occult deposition in water and nutrient supply to the Strandveld is the foliar chemistry of this vegetation. Concentrations of P, Na, Mg, and Ca were all higher in Strandveld vegetation (S2 Table) than Fynbos components of the CFR [71]. In contrast, Strandveld foliar K was substantially lower than would be expected based on deposition, soil K concentrations and in comparison to other CFR sites [71]. This is possibly because the relatively high soil Na competitively inhibits K uptake at the root plasmalemma, which can lead to K deficiency in plants [72] and loss of K through leaching. The foliar N:P ratios of non-N_2_-fixing (i.e. excluding *M*. *cordifolia*; [4]) Strandveld species were relatively low (5.8), consistent with low concentrations of soil N relative to P (Table 4). Our measures of nutrient loss in leaf litter of the dominant woody species provided an estimate of the potential contribution of HP to ecosystem nutrient demand that indicated that HP could meet the demands for N, P, K and Ca. Although it is unlikely that the plants depend extensively on HP directly, this input of nutrients relative to ecosystem consumption perhaps explains why relatively young and (recently) mobile dune sands have relatively high concentrations of some nutrients.

HP is likely deposited directly both on leaves and on soil surfaces, contributing nutrients and moisture. The vegetation of the area may intercept moisture and aerosols that drip from leaves into the soil, resulting in the observed enrichment of soils around vegetation clumps [2]. We did not measure this throughfall of water and nutrients, but it is potentially an important component of nutrient deposition. Occult deposition may also play an additional important role in these ecosystems by providing both moisture and nutrients through foliar deposition. Small leaved Strandveld species intercepted the most moisture on a per area basis. *A*. *imbricata* is a low-stature perennial with an abundance of fine narrow leaves, while *M*. *muricata* has small hairy leaves. These leaf traits may contribute to retention of depositional moisture by these species, as previously suggested [42]. This intercepted moisture may be taken up directly through the leaf surfaces [39, 11] or indirectly from the soil as fog drip and stem flow [9, 11]. Foliar uptake of nutrients is common, depending on the nutrient forms and concentrations [40, 41] and, the duration of moisture on the leaf [9]. As with water retention, the smaller-leaved Strandveld species had higher concentrations of nutrients deposited on the leaves per unit surface area compared to the other species, indicating that nutrient deposition on leaf surfaces at least partially scales with water retention by the leaves. The uptake of ^15^N-labelled glycine, NO_3_^-^ and NH_4_^+^ and Li (as a tracer for K; [73]) also demonstrates that the plants have the capacity to absorb these nutrients through their leaves. This shows the capacity for organic and inorganic N uptake by all species, but the reasons for the differences are likely to be complicated and outside the scope of this study. The variations in uptake of glycine, NO_3_^-^ and NH_4_^+^ between species may be due to differences in leaf properties such as size, surface properties (e.g. trichomes, waxiness), cuticular conductance, stomatal conductance and/or capacity to transport the N into the leaf tissue [74, 75, 76, 77]. The uptake of ^15^N-glycine is particularly important considering the high proportion of DON in HP. Direct leaf uptake of DON would allow plants to avoid competitive interactions with other plants and microbes in the rooting zone and may serve as a competitive advantage in these ecosystems [78]. Considering the significant potential contribution of HP to nutrient deposition, it is likely that the vegetation participates in both the direct interception of the nutrients, as well as increasing throughfall deposition to the soil. Furthermore, selective plant uptake of some nutrients may enable retention of these nutrients, whereas those not taken up are more susceptible to leaching.

## Conclusions

The evidence presented indicates that deposition may be an important source of nutrients for Strandveld ecosystems. The flux of nutrients in rainfall in the wet winter months combined with inputs of HP, especially during the drier summers, suggests that atmospheric nutrient deposition may play a potentially important, year-around, role in plant nutrition at these sites. The depositional nutrient load and the ability of the vegetation to intercept and take up foliar deposition may explain why this vegetation is distinct floristically from neighboring Fynbos vegetation that occurs on less nutrient rich soils [79]. Although the relative lack of fire in Strandveld may also be important in determining vegetation structure, floristic characteristics and nutrition, we suggest that the name of this vegetation, meaning “beach vegetation” in Afrikaans, adequately describes the reason for the existence of this particular vegetation.

## Supporting Information

S1 Dataset Excel file with raw data. (XLSX)

S1 Fig A diagram of the apparatus used to trap horizontal precipitation in the field. (TIFF)

S2 Fig Correlation between horizontal precipitation (HP) and site elevation. The distances (km) from coast along the transect are shown alongside the average of the 2011 monthly HP deposition rate (mean ± SE, n = 12). The regression line was fitted to all data, but excluding the coastal site (0.1 km).
(TIFF)

S3 Fig Rates of leaf litter production of the sampled plant species. Bars represent mean ± SE for litterfall production in each species: *M*. *cordifolia* (n = 3), *C*. *monelifera* (n = 3), *S*. *glauca* (n = 9), *S*. *lucida* (n = 5), *A*. *imbricate* (n = 8) and *M*. *muricata* (n = 12). Significant differences (*P* < 0.05) between the species were determined using Tukey post-hoc tests following a one-way ANOVA, and are represented by different letters.
(TIFF)

S1 Table Comparison of the average monthly concentrations of various nutrients in horizontal precipitation measured at sites in the study area (mean ± SE; mg L^-1^ month^-1^, n = 7) with that at the coastal site closest to the ocean (0.1 km inland). (DOCX)

S2 Table Concentrations of nutrients measured in green and senesced foliar tissues of the sampled species. Concentrations (mean ± SE) for elements measured in green tissue (G) and senesced tissue (S) for *M*. *cordifolia*, *C*. *monelifera*, *S*. *glauca*, *S*. *lucida*, *A*. *imbricata* and *M*. *muricata* sampled within the study site in Nov 2011. The overall averages were calculated over the six species. The percentage of each element remaining in the senesced leaves is also shown.
(DOCX)
