# Supplementary figures and images for: The Contribution of Occult Precipitation to Nutrient Deposition on the West Coast of South Africa
Source: PLoS One. 2015 May 27;10(5):e0126225. doi: 10.1371/journal.pone.0126225 (PMC4446095; doi:10.1371/journal.pone.0126225)

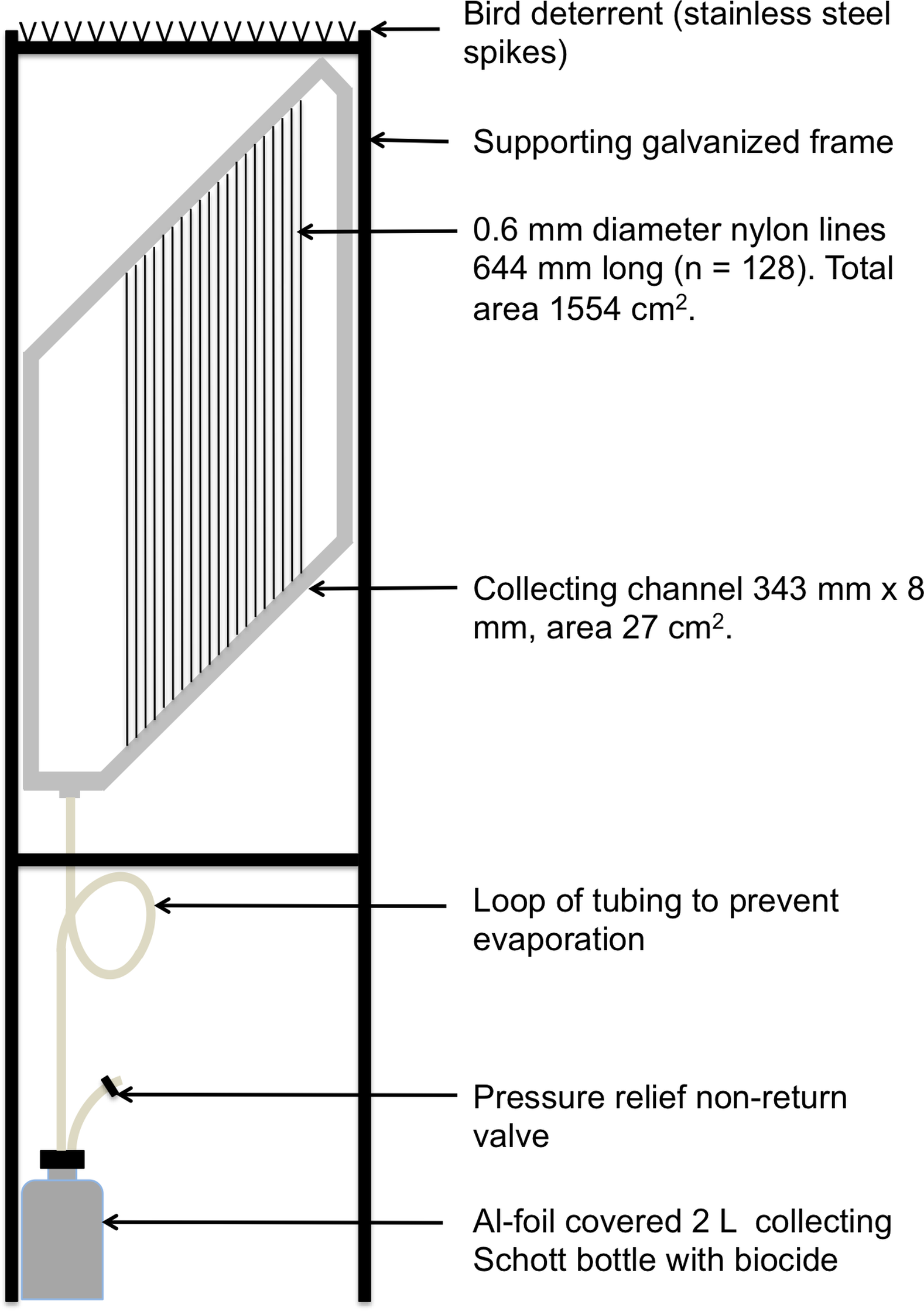

Supplement: S1 Fig — (TIFF) [file pone.0126225.s002.tiff]

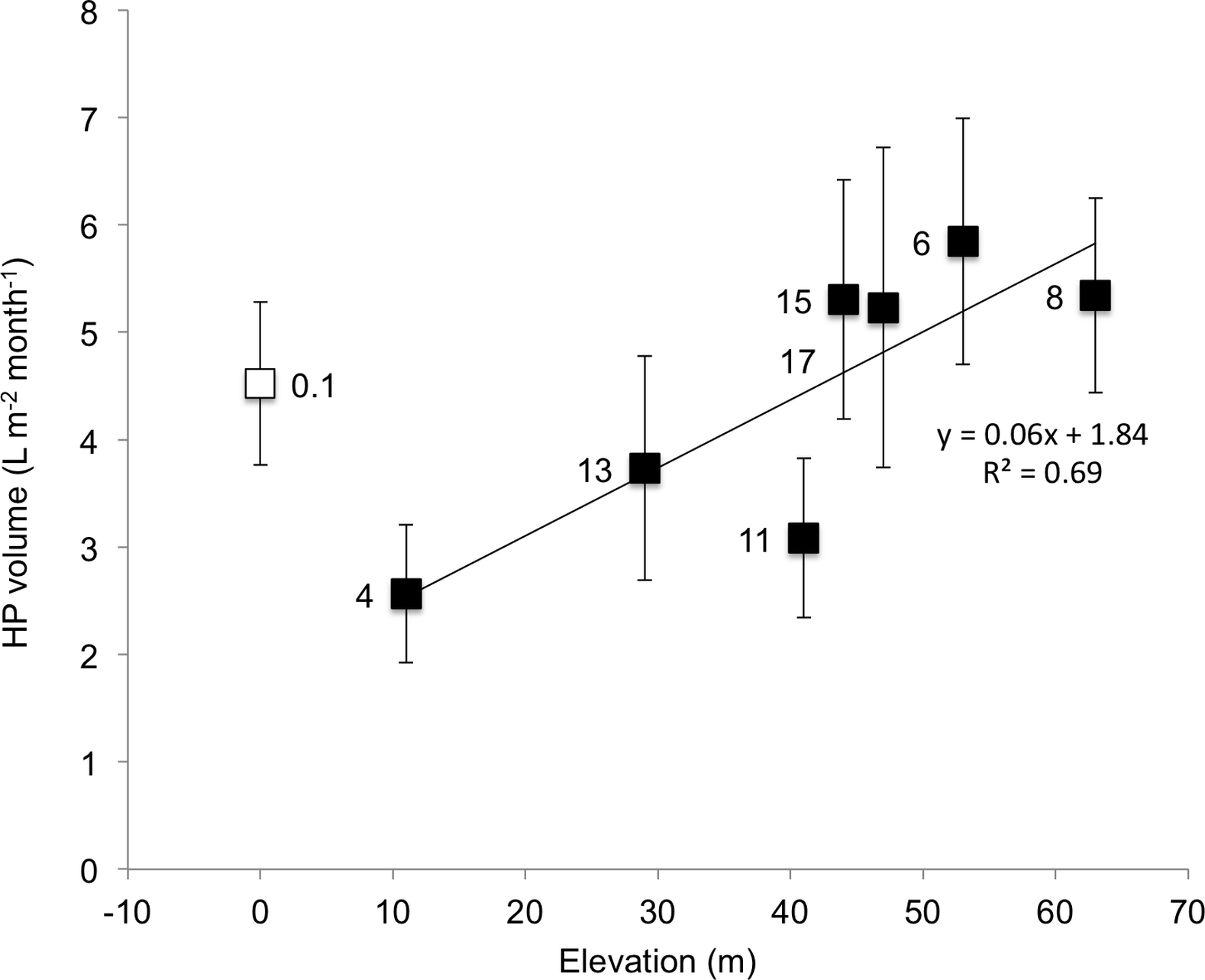

Supplement: S2 Fig — The distances (km) from coast along the transect are shown alongside the average of the 2011 monthly HP deposition rate (mean ± SE, n = 12). The regression line was fitted to all data, but excluding the coastal site (0.1 km). (TIFF) [file pone.0126225.s003.tiff]

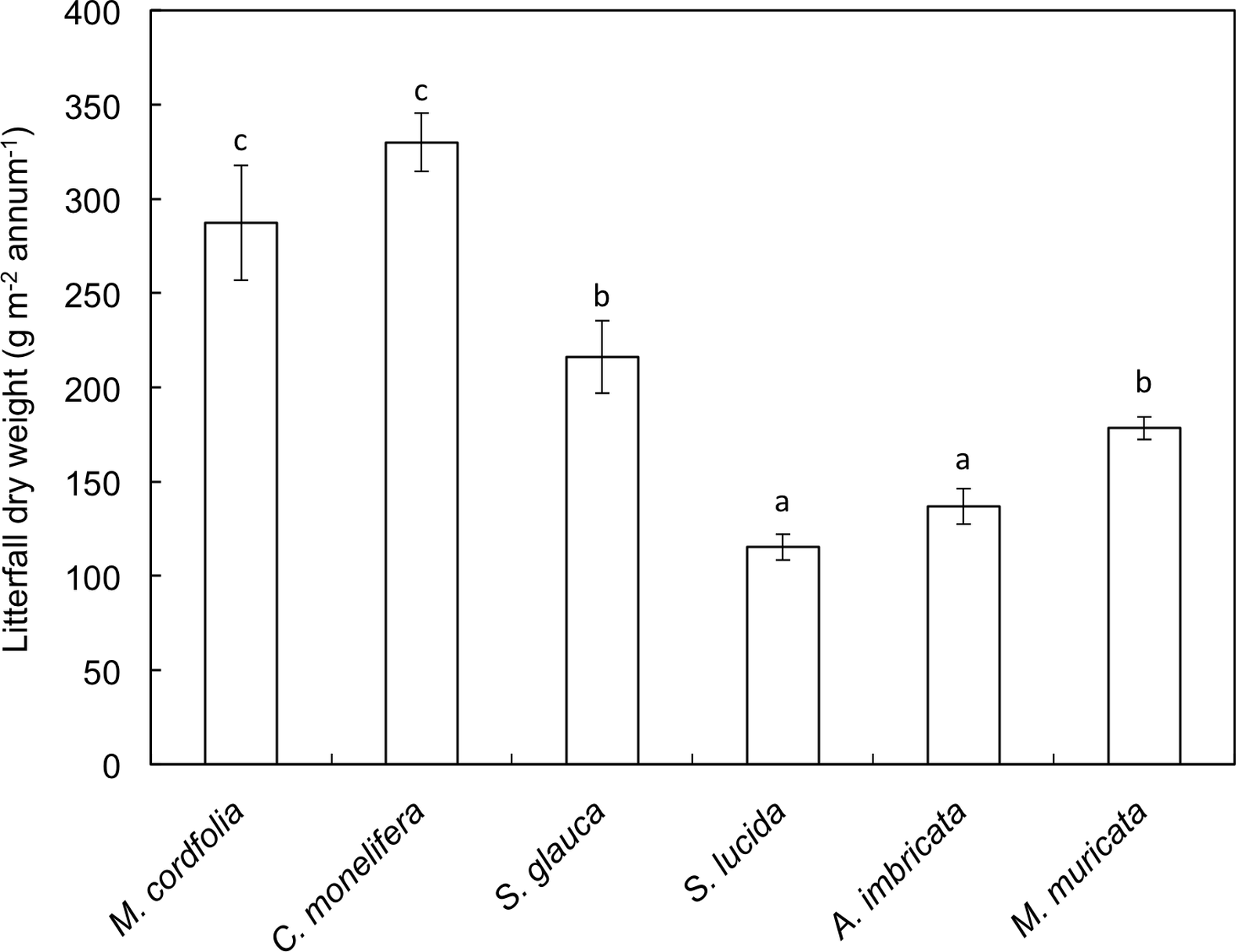

Supplement: S3 Fig — Bars represent mean ± SE for litterfall production in each species: M. cordifolia (n = 3), C. monelifera (n = 3), S. glauca (n = 9), S. lucida (n = 5), A. imbricate (n = 8) and M. muricata (n = 12). Significant differences (P < 0.05) between the species were determined using Tukey post-hoc tests following a one-way ANOVA, and are represented by different letters. (TIFF) [file pone.0126225.s004.tiff]
