# Supplementary material for: The Contribution of Occult Precipitation to Nutrient Deposition on the West Coast of South Africa
Source: PLoS One. 2015 May 27;10(5):e0126225. doi: 10.1371/journal.pone.0126225 (PMC4446095; doi:10.1371/journal.pone.0126225)
Supplement: S1 Table — (DOCX) [file pone.0126225.s005.docx]

| **Element** | **Coastal site** | **Average of inland sites** |
| --- | --- | --- |
| total N | 60 | 27±2 |
| NO_3_^–^ | 3.2 | 3.4±0.5 |
| DON | 41 | 23±2 |
| Total P | 5.8 | 0.4±0.1 |
| SRP | 0.93 | 0.14±0.03 |
| Mg | 2127 | 76±16 |
| Ca | 631 | 41±4 |
| Na | 18709 | 640±141 |
| K | 659 | 23±4 |
| Si | 16 | 0.7±0.2 |
| Mn | 0.057 | 0.050±0.004 |
| Fe | 0.8 | 0.06±0.02 |
| Al | 1.03 | 0.09±0.03 |
