# Supplementary material for: The Contribution of Occult Precipitation to Nutrient Deposition on the West Coast of South Africa
Source: PLoS One. 2015 May 27;10(5):e0126225. doi: 10.1371/journal.pone.0126225 (PMC4446095; doi:10.1371/journal.pone.0126225)
Supplement: S2 Table — Concentrations (mean ± SE) for elements measured in green tissue (G) and senesced tissue (S) for M. cordifolia, C. monelifera, S. glauca, S. lucida, A. imbricata and M. muricata sampled within the study site in Nov 2011. The overall averages were calculated over the six species. The percentage of each element remaining in the senesced leaves is also shown. (DOCX) [file pone.0126225.s006.docx]

| **Species name** |  | **Total N**  **mg g^-1^** | **Total P**  **mg g^-1^** | **K**  **mg g^-1^** | **Ca**  **mg g^-1^** | **Na**  **mg g^-1^** | **Mg**  **mg g^-1^** | **Fe**  **μg g^-1^** | **Mn**  **μg g^-1^** | **n** |
| --- | --- | --- | --- | --- | --- | --- | --- | --- | --- | --- |
| *M. cordfolia* | G | 16.0±0.2 | 0.49±0.23 | 1.66±0.51 | 31±3 | 3.9±0.8 | 2.3±0.1 | 215±23 | 83±13 | 3 |
|  | S | 10.6±0.2 | 0.10±0.01 | 0.24±0.00 | 1.6±0.1 | 0.06±0 | 0.2±0 | 17±2 | 4.2±0.5 | 3 |
|  | % | 66±2 | 28±10 | 17±5 | 5±1 | 1.6±0.4 | 10±1 | 8±1 | 6±2 | 3 |
| *C. monelifera* | G | 14.0±2.8 | 0.72±0.08 | 1.96±0.46 | 22±1 | 27.3±7 | 6.2±1.1 | 165±33 | 5±1 | 3 |
|  | S | 7.3±2.2 | 0.14±0.02 | 0.28±0 | 2.6±0.1 | 0.07±0 | 0.6±0.1 | 7±1 | 0.7±0.1 | 3 |
|  | % | 50±5 | 19±0 | 16±4 | 12±1 | 0.3±0.1 | 11±2 | 5±1 | 15±2 | 3 |
| *S. glauca* | G | 8.9±0.9 | 2.18±0.39 | 3.05±0.64 | 26±2 | 4.0±0.7 | 3.2±0.3 | 76±16 | 9±2 | 9 |
|  | S | 5.6±0.5 | 0.16±0.01 | 0.26±0.01 | 1.4±0.1 | 0.06±0 | 0.2±0 | 7±1 | 0.6±0.1 | 9 |
|  | % | 69±5 | 10±2 | 11±2 | 6±1 | 2.1±0.4 | 9±2 | 12±2 | 8±1 | 9 |
| *S. lucida* | G | 11.5±2.5 | 2.08±0.32 | 2.77±0.39 | 29±3 | 4.7±0.9 | 4.3±0.5 | 53±7 | 4±1 | 5 |
|  | S | 5.9±0.3 | 0.17±0.00 | 0.24±0.02 | 2.3±0.5 | 0.07±0 | 0.4±0.1 | 9±1 | 0.6±0.1 | 5 |
|  | % | 70±13 | 9±1 | 10±2 | 8±1 | 1.6±0.2 | 10±2 | 18±3 | 18±5 | 5 |
| *A. imbricata* | G | 9.9±0.7 | 1.4±0.38 | 2.7±0.52 | 30±7 | 2.0±0.4 | 2.0±0.5 | 249±66 | 10±2 | 8 |
|  | S | 5.6±0.4 | 0.13±0.01 | 0.26±0.01 | 1.8±0.2 | 0.06±0.00 | 0.2±0 | 13±3 | 1.2±0.4 | 8 |
|  | % | 57±7 | 15±3 | 13±2 | 8±2 | 4.7±1.4 | 16±4 | 8±3 | 12±2 | 8 |
| *M. muricata* | G | 9.1±0.4 | 0.83±0.15 | 2.39±0.36 | 19±2 | 11.0±3.6 | 3.2±0.6 | 307±26 | 13±3 | 12 |
|  | S | 6.2±0.3 | 0.11±0.01 | 0.32±0.05 | 1.6±0.2 | 0.07±0.01 | 0.2±0 | 44±20 | 0.7±0.1 | 12 |
|  | % | 68±3 | 18±4 | 24±11 | 12±4 | 2.3±0.7 | 9±3 | 22±11 | 7±1 | 12 |
| Average | G | 11.6±0.5 | 1.28±0.05 | 2.42±0.04 | 26±1 | 8.8±1.1 | 3.5±0.1 | 178±8 | 21±2 | 6 |
|  | S | 6.9±0.3 | 0.13±0.00 | 0.27±0.01 | 1.9±0.1 | 0.07±0 | 0.3±0 | 16±3 | 1.3±0.1 | 6 |
|  | % | 63±2 | 16±1 | 15±1 | 8±0 | 2.1±0.2 | 11±0 | 12±2 | 11±1 | 6 |
